# Comparative Long-Wave Infrared Laser-Induced Breakdown Spectroscopy Employing 1-D and 2-D Focal Plane Array Detectors

**DOI:** 10.3390/s23031366

**Published:** 2023-01-26

**Authors:** Clayton S.-C. Yang, Feng Jin, Sudhir Trivedi, Uwe Hommerich, Alan C. Samuels

**Affiliations:** 1 Brimrose Corporation of America, Baltimore, MD 21152, USA; 2 Department of Physics, Hampton University, Hampton, VA 23668, USA; 3 Edgewood Chemical Biological Center, Aberdeen Proving Ground, MD 21010, USA

**Keywords:** laser-induced breakdown spectroscopy, laser-induced plasma, long-wave infrared spectroscopy, atomic emission, molecular emission

## Abstract

Long-wave infrared (LWIR) emissions of laser-induced plasma on solid potassium chloride and acetaminophen tablet surfaces were studied using both a one-dimensional (1-D) linear array detection system and, for the first time, a two-dimensional (2-D) focal plane array (FPA) detection system. Both atomic and molecular infrared emitters in the vicinity of the plasma were identified by analyzing the detected spectral signatures in the infrared region. Time- and space-resolved long-wave infrared emissions were also studied to assess the temporal and spatial behaviors of atomic and molecular emitters in the plasma. These pioneer temporal and spatial investigations of infrared emissions from laser-induced plasma would be valuable to the modeling of plasma evolutions and the advances of the novel LWIR laser-induced breakdown spectroscopy (LIBS). When integrated both temporally (≥200 µs) and spatially using a 2-D FPA detector, the observed intensities and signal-to-noise-ratio (SNR) of single-shot LWIR LIBS signature emissions from intact molecules were considerably enhanced (e.g., with enhancement factors up to 16 and 3.76, respectively, for a 6.62 µm band of acetaminophen molecules) and, in general, comparable to those from the atomic emitters. Pairing LWIR LIBS with conventional ultraviolet–visible–near infrared (UV/Vis/NIR) LIBS, a simultaneous UV/Vis/NIR + LWIR LIBS detection system promises unprecedented capability of in situ, real-time, and stand-off investigation of both atomic and molecular target compositions to detect and characterize a range of chemistries.

## 1. Introduction

In situ detection and identification of trace hazardous compounds, including chemical warfare agents and toxic industrial materials, on operational surfaces is vital for reconnaissance and early warning operations in various security and defense applications. Hazardous surface contamination presents a serious threat both to civilians and military personnel. Adequate defense against these dangerous surface contaminations will require the rapid detection and identification of both known and unknown chemicals. Current state-of-the-art techniques for the detection of chemical contamination on operational surfaces are an often time- and resource-intensive reconnaissance mission limited to contact sampling and time-consuming colorimetric or molecular analysis that places personnel or detection devices in direct contact with the hazardous materials.

Recent advances in laser-based optical spectroscopy demonstrate the effectiveness of non-contact methods for the remote sensing of chemicals deposited on surfaces [1]. For example, ultraviolet Raman spectroscopy demonstrates potential for non-contact standoff detection of hazardous materials on surfaces, but some great challenges remain. For remote optical reflectance techniques, such as Raman spectroscopy, the spectral signature signals are usually very weak and the accuracy of the surface contamination measurements are frequently influenced by the shape, roughness, composition irregularity of the target surfaces, and complex background emissions (e.g., fluoresce from the surface) [2]. An ideal optical technique in the reconnaissance mission should have intense signal responses and be affected very little by the surface shape, roughness, and composition irregularity. This technology should also be able to provide in situ, real-time, or near-real-time analysis, regardless of the physical and optical properties of the sample surface, without the need for sample preparations. Considering these requirements, a recently developed optical technology, simultaneous UV/Vis/NIR + LWIR laser-induced breakdown spectroscopy (LIBS), offers great promise for in situ and real-time surface contaminant identification in reconnaissance applications [3,4].

Laser-induced breakdown spectroscopy (LIBS) utilizes laser-induced plasma (LIP) for direct sampling to provide rapid chemical detection and identification without the need for sample preparation. In essence a point-and-shoot atomic spectroscopy technique, LIBS can acquire a UV/Vis/NIR (200–1000 nm) spectrum of multi-elemental emission signatures with relative reasonable detection limits, typically in the range of 10–1000 ppm, from all the elements in the periodic table within microseconds [5]. It has been used in the past few decades for various terrestrial applications, such as environmental characterization, mineral geology, and military survey [6,7]. Although LIBS is very sensitive to the elemental composition of the target materials, it is not sensitive to the bonding and molecular structures of the sample materials under study. Due to their elemental composition similarities, the detection and identification of organic materials presents particular challenges for LIBS-based chemical analyzers.

Recent studies of mid-wave to long-wave IR (MWIR to LWIR) emissions (2–12 µm) from laser-induced plasmas discovered various intact target molecules existing in the vicinity of the plasma [4,8]. Those molecular gases in the vicinity of the vapor–plasma plume were thermally excited by the hot plasma and emitted vibration–rotation spectral signatures in the mid-IR (MWIR to LWIR) region which could be readily used for molecular identification. Based on those discoveries, an innovative LWIR LIBS spectroscopy, sometimes referred to as laser-induced thermal emission (LITE), was recently developed [1,2,3,4]. In LWIR LIBS, the molecular structures of constituents are revealed by spectrally analyzing the emission profile from a laser-induced plasma generated on the target surface using mid-IR spectroscopy. To the best of our knowledge, LWIR LIBS is, to-date, the only vibrational–rotational mid-IR emission spectroscopy technique that offers the same instrumental and analytical advantages of both conventional UV/Vis/NIR LIBS and Raman spectroscopy in on-site field applications [1,2,3,4,9]. One of the greatest advantages of this new spectroscopy is its simplicity to pair with a UV/Vis/NIR LIBS spectrometer. Placed side-by-side, the conventional UV/Vis/NIR LIBS spectrometer captures the ionic, atomic emissions of laser-induced plasma generated on the target surface in the UV/Vis/NIR region, while the LWIR LIBS spectrometer acquires the molecular emissions of the same plasma plume in the MWIR to LWIR region simultaneously. With all the intrinsic advantages of conventional LIBS, simultaneous UV/Vis/NIR + LWIR LIBS offers unprecedented capability of in situ, real-time, and stand-off investigation of both atomic and molecular compositions of surfaces, coatings, and layers below the surface.

In this work, standoff (1 m) simultaneous UV/Vis/NIR LIBS + LWIR LIBS studies of potassium chloride and an organic compound, acetaminophen, were presented. Characteristic emission bands of excited atoms and molecules were detected and identified in both UV/Vis/NIR and LWIR emissions from laser-induced plasma. Standoff LWIR LIBS emissions were studied using both a one-dimensional (1-D) linear array detection system (Section 3.1 and Section 3.2) and, for the first time, a two-dimensional (2-D) focal plane array detection system (Section 3.3). By detecting and characterizing organic compounds from a standoff distance, LWIR LIBS showed UV/Vis/NIR LIBS comparable detection limits and a high degree of surface condition tolerances. Time- and space-resolved LWIR emissions were also studied to assess the temporal and spatial behaviors of both atomic and molecular emitters in the plasma.

## 2. Materials and Methods

**Figure 1:**
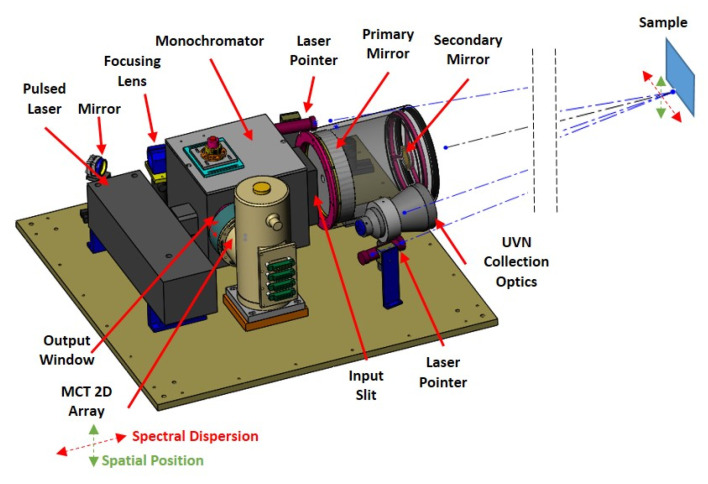
Bench-top simultaneous UV/Vis/NIR (UVN) + LWIR LIBS detection system.

The details of the experimental setup, a lab-based bench-top simultaneous UV/Vis/NIR + LWIR LIBS prototype spectrometer, and calibration procedures have been discussed in detail in previous studies [2,4]. This bench-top detection system can remotely probe the target from a distance from 0.1 up to 6 m utilizing a compact Q-switch Nd:YAG laser excited (1064 nm), fixed grating and mercury–cadmium–telluride (MCT) linear detector array (1 × 332 detector elements, 50 × 50 µm pitch size, integrated with a readout integrated circuit (ROIC)) based spectrometer for LWIR LIBS measurements, and a grating + CCD-based UV/Vis/NIR spectrometer covering 200–1000 nm for UV/Vis/NIR LIBS measurements. This UV/Vis/NIR + LWIR LIBS system simultaneously captured emission signatures from atomic, diatomic, and complex molecular target species from laser-induced plasma generated on the sample target surface in both the UV/Vis/NIR (350–1000 nm) and LWIR (5.6–10 µm) spectral range. These spectral signatures can be readily used for target identification and characterization. In this study (as shown in Figure 1), a two-dimensional (2-D) MCT focal plane array (FPA) detector (PhaseTech MCT 2-D array with a pixel format of 128 × 128 and 40 × 40 µm pitch size, integrated with ROIC) was tested for the first time in LWIR LIBS measurements, besides the typical linear MCT array detector used in previous studies and in Section 3.1 and Section 3.2 of the current work [1,2,3,4,8,9,10]. Sharing the same excitation and collection optics setup of the bench-top detection system (Figure 1), the spectral resolutions for both 1-D and 2-D detection measurements were about 76 nm [3].

KCl and acetaminophen were selected to be the sample compounds in this 1-D and 2-D comparison study for their multiple intense and well-separated emission signatures in the LWIR region between 6 and 9 µm [3]. For LWIR LIBS-based sensor developments, these two chemical compounds are among the best candidates for calibration and reference samples. In this study, the solid sample tablets were prepared by pressing pure substance powders (e.g., KCl and acetaminophen) and powder-mixtures (e.g., acetaminophen/Al_2_O_3_) with a hydraulic press (~7 tons of pressure) for 5 s. The solid sample tablets were vertically mounted on a sample stage which was placed one meter away from the front end of the collection optic telescope and probed in an ambient air atmosphere. If not stated specifically, the exciting laser pulse energy of the presented work was set to 164 mJ, corresponding roughly to an energy density of 4.48 GW/cm^2^ on the sample surface, with the delay time set to 3 µs (integration time: 10 μs) for UV/Vis/NIR measurements and 20 µs (integration time: 40 μs) for LWIR measurements by the linear MCT array detector. All the LIBS emission spectra captured by the linear MCT array detector presented in Section 3.1 and Section 3.2 of this work were acquired by averaging four single-shot spectra on the same spot on the sample surface for a better repeatability and signal-to-noise ratio (SNR) [2,3,8]. Estimated in six repeated measurements, the relative standard deviations (RSDs) of LWIR emission features between 6 and 9 µm were under 13.76% in all averaged four-single-shot spectra in this study (except in the 0.1% acetaminophen/Al_2_O_3_ mixture spectrum which ranged from 14.9% to 43.4%). These reasonably moderate RSDs [11,12,13] allowed us to perform better qualitative analysis of the emission features for target classification and identification as we have done in previous studies [2,3,4,8]. All the LIBS emission spectra captured by the 2-D FPA MCT detector presented in this work were acquired by just a single-shot on the solid sample surface. The signal-to-noise ratios (SNRs) of the single-shot 1-D and 2-D LWIR spectra in Section 3.3 were evaluated using eight independently measured single-shot spectra for each KCl and acetaminophen sample tablet. To reduce the effect of the intensity fluctuation on noise estimation, normalized spectra were used in SNR estimation.

## 3. Results and Discussion

### 3.1. UV/Vis/NIR LIBS and LWIR Emission Signatures from Laser-Induced Plasma

**Figure 2:**
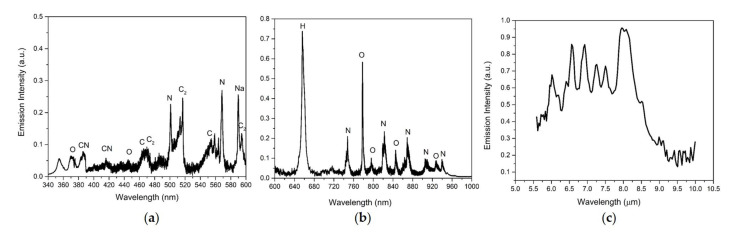
UV/Vis/NIR (**a**,**b**) + LWIR (**c**) LIBS spectrum of solid acetaminophen tablets.

**Figure 3:**
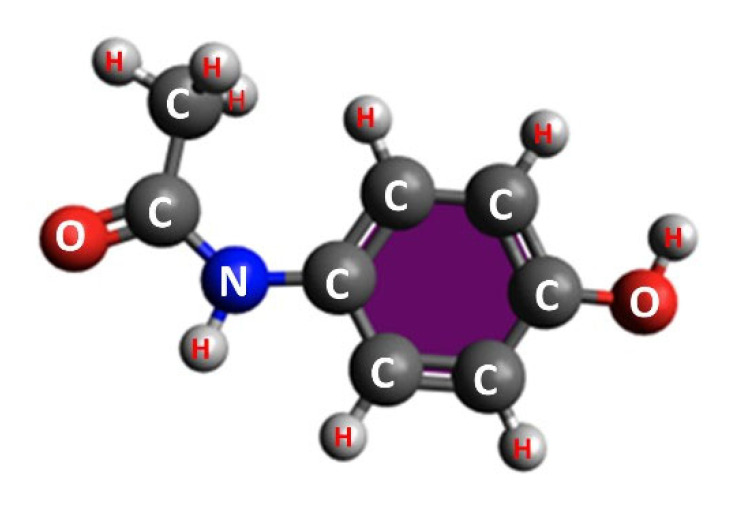
Optimized structure of the acetaminophen molecule calculated with DFT(BP86)/def2-TZVP using ORCA.

Figure 2 shows the simultaneous UV/Vis/NIR + LWIR LIBS spectra of a pure acetaminophen (paracetamol) tablet under an ambient air atmosphere. Acetaminophen (C_8_H_9_NO_2_ Figure 3) is an organic compound which is widely used as a pharmaceutical pain reliever. In the UV/Vis/NIR LIBS spectrum of acetaminophen (Figure 2a,b), emission signatures due to electronic transitions of typical atomic and di-atomic constituents of an organic compound could be observed: C atoms at 422 nm, 466 nm, and 553 nm; H atoms at 383 nm, 410 nm, 486 nm, and 656 nm; N atoms at 451 nm, 500 nm, 747 nm, 822 nm, 868 nm, 905 nm, and 939 nm; O atoms at 375 nm, 444 nm, 777 nm, 795 nm, 845 nm, and 926 nm; a di-atomic CN molecule at 388 nm and 415 nm; and a di-atomic C_2_ molecule at 467–473 nm and 513–516 nm [14,15]. In the LWIR LIBS spectrum of pure acetaminophen, several intense molecular vibrational emission features could be readily observed between 5.6 and 10 µm. To elucidate the nature of those intense LWIR emission bands, ab initio quantum chemistry calculations were carried out. Quantum chemical approximation using density function theory provided good accuracy benchmarks for evaluations of molecular behaviors in the gas phase and in solution [16,17]. DFT calculations of acetaminophen molecular structures and vibration characters using the BP86 functional with the def2-TZVP basis set and the def2/J auxiliary basis set in ORCA software package [18] gave the best agreements with LWIR LIBS results without the need of any scaling factor. The vibrational mode assignments of the LWIR LIBS features of pure acetaminophen are listed in Table 1 and were mostly comparable to previous vibrational spectroscopy studies of acetaminophen [19,20]. From the good agreements between the DFT calculations of molecular vibration modes and characteristic frequencies and the LIBS measurements, the LWIR LIBS spectrum of pure acetaminophen is apparently dominated by the sharp and intense vibrational signature emissions of intact acetaminophen molecules. The relative intensities of the N, O, and CN features in the UV/Vis/NIR LIBS spectrum were greatly influenced by the interference from the emissions of ambient air (with abundant nitrogen and oxygen). This possible source of errors for qualitative and quantitative analysis of organic compounds presents great challenges for fast organic identification in field applications using UV/Vis/NIR LIBS-based sensors. In contrast, the LWIR vibrational signatures of organic compounds observed from laser-induced acetaminophen plasma were perturbed much less by the ambient N_2_ and O_2_. LIBS measurements in the LWIR proved to be able to yield additional molecular structure information of the target substances, which complements elemental results obtained from conventional UV/Vis/NIR LIBS measurements for accurate real-time identification and classification.

**Figure 4:**
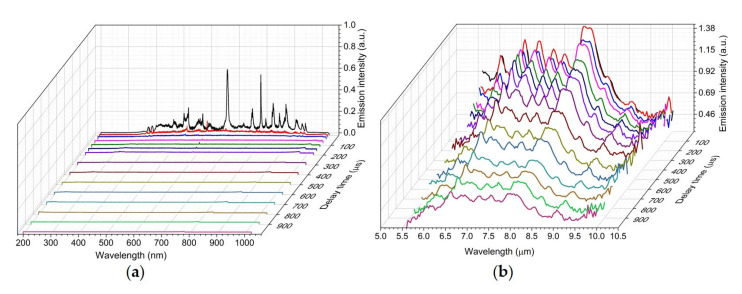
Simultaneous UV/Vis/NIR (**a**) + LWIR (**b**) LIBS spectrum of acetaminophen tablet measured at a delay time of 1 µs, 20 µs, 40 µs, 80 µs, 120 µs, 160 µs, 200 µs, 300 µs, 400 µs, 500 µs, 600 µs, 700 µs, 800 µs, 900 µs, and 1000 µs.

**Figure 5:**
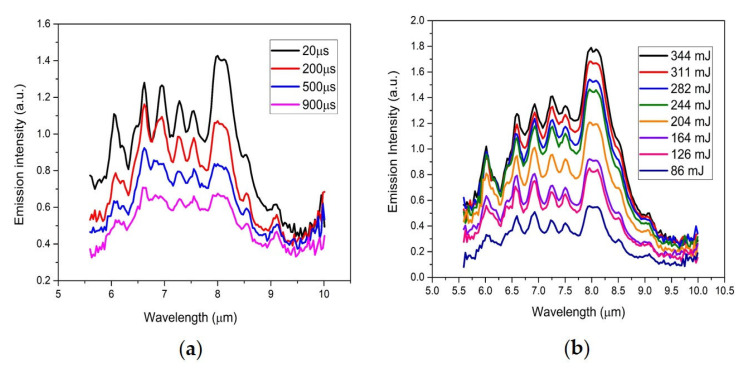
(**a**) LWIR LIBS spectrum of an acetaminophen tablet measured at a delay time of 20 µs, 200 µs, 500 µs, and 900 µs; (**b**) LWIR LIBS spectrum of an acetaminophen tablet measured at a delay time of 20 µs with different excitation laser pulse energies.

The temporal dependence of the UV/Vis/NIR and LWIR emissions of the laser-induced plasma measured simultaneously on pure acetaminophen a solid tablet are shown in Figure 4. The electronic emissions from atoms and small diatomic molecules in the UV/Vis/NIR LIBS spectrum quickly disappeared after 20 µs. The molecular vibrational emissions of intact acetaminophen molecules persisted for hundreds of micro-seconds in the LWIR LIBS spectrum. All the major vibrational signature bands of acetaminophen molecules were still clearly identifiable at 1 millisecond after the plasma initiation. The existence of vibrationally excited molecules apparently lasted much longer than their electronically excited atomic and di-atomic molecular counterparts in the laser-induced acetaminophen vapor–plasma plume. Although the spectral signatures of those vibrationally excited molecules were evidently observed throughout hundreds of micro-seconds, the relative intensities of those signature bands changed with time. For example, the CH_3_ deformation band at 6.95 µm was much more prominent at an early time but decayed much faster than the other CH_3_ deformation band at 6.83 µm (Figure 5a). A similar trend was observed at the temporal evolution of laser-induced methanol plasma [9] due to the faster decays of IR-radiating molecular breakdown fragments of methanol molecules such as C_2_H_2_, CH_2_O, and CH_4_. Quite a few CH-containing compounds have their CH_3_ or CH_2_ deformation modes located in this 6.95 µm range [21]. Therefore, some of the molecular breakdown fragments or products of acetaminophen could contribute to the faster decaying emission features observed around 6 µm, 6.95 µm, and 8 µm.

The power dependence of the LWIR emissions from laser-induced acetaminophen plasma is shown in Figure 5b. The LWIR LIBS spectra of laser-induced acetaminophen plasma are dominated by emission features of acetaminophen molecules with their overall intensities growing at higher surface photon energy density. All eleven vibrational signatures of intact acetaminophen molecules (Table 1) were persistently present in the LWIR LIBS spectra as excitation laser energy increasing from 86 mJ per pulse (surface energy density ~2.35 GW/cm^2^) to 344 mJ per pulse (surface energy density ~12 GW/cm^2^). Even with a vastly elevated plasma temperature at higher excitation energy [22], there are still sufficient hot, intact acetaminophen molecules in the vicinity of the vapor–plasma plume radiating in the infrared region. The overall line shape of the LWIR LIBS acetaminophen spectrum varied with excitation power. The faster decaying emission features (e.g., bands around 6 µm, 6.95 µm, and 8 µm) also grew more prominent with excitation power. This seems to support the existence of some molecular breakdown fragments or products contributing emissions near these wavelength ranges. In addition, while the intensity of the 8 µm band grew nearly linearly with the excitation power from 86 mJ to 344 mJ, the intensity of the 6 µm band exhibited the onset of saturation for pulse energies above ~244 mJ. More detailed studies of the LIBS signal intensity versus excitation energy and the underlying mechanisms are ongoing and will be published in the near future.

**Figure 6:**
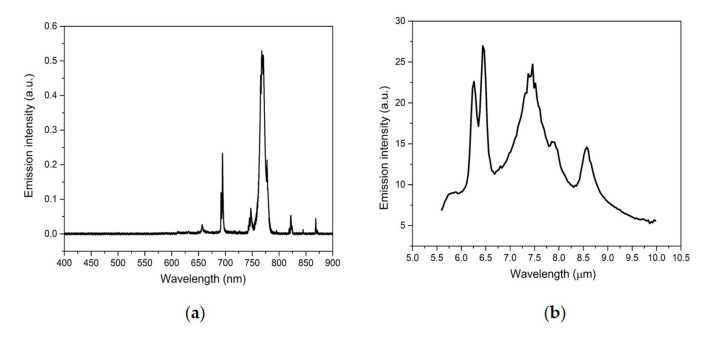
Simultaneous UV/Vis/NIR (**a**) + LWIR (**b**) LIBS spectrum of a solid KCl tablet.

**Figure 7:**
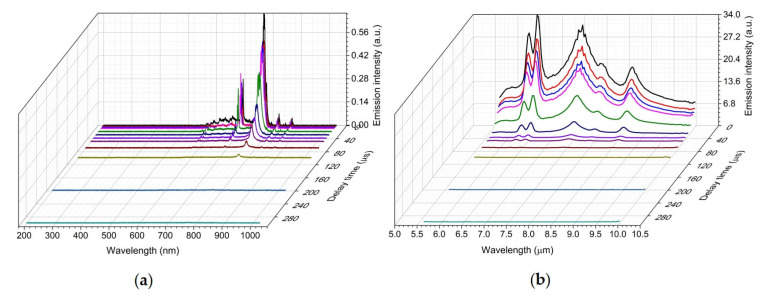
Simultaneous UV/Vis/NIR (**a**) + LWIR (**b**) LIBS spectrum of an acetaminophen tablet measured at a delay time of 1 µs, 3 µs, 6 µs, 10 µs, 20 µs, 30 µs, 40 µs, 50 µs, 70 µs, 100 µs, 200 µs, and 300 µs.

Besides the signature emissions from vibrationally excited intact sample molecules, we also observed atomic signature emission bands from the laser-induced plasma in the LWIR region. Intense emission bands were observed from laser-induced plasma on the surface of a KCl tablet in both the UV/Vis/NIR and LWIR regions (Figure 6). The intense visible and NIR emission peaks at 691 nm, 694 nm, 766 nm, and 770 nm can be attributed to the de-excitation of neutral potassium K atoms via the electronic transitions of 6S_1/2_ to the first excited state doublet 4P_1/2_ and 4P_3/2_ and the electronic transitions of the first excited state doublet 4P_1/2_ and 4P_3/2_ back to the ground state 4S_1/2_ [23]. KCl is a diatomic molecule with a fundamental vibrational wavelength (~35 µm) in the far infrared region [24]. Therefore, KCl could be used as a transmission window material in mid-IR spectroscopies. However, interestingly, the LIBS spectrum of KCl in the LWIR region was not featureless. Intense and distinct LWIR atomic emission signatures centered at 6.23 μm, 6.44 μm, 7.43 μm, 7.89 μm, and 8.53 μm were readily observed in the LIBS spectrum of KCl. These LWIR emission features could be attributed to the inter-high-excited-states transitions of neutral potassium atoms: 6P_1/2_ → 4D_3/2_, 6P_1/2_ → 6S_1/2_, 6H → 5G, 7S_1/2_ → 6P_3/2_, and 5D_5/2_ → 6P_3/2_, respectively [3,25]. The UV/Vis/NIR and LWIR emissions from laser-induced plasma measured simultaneously on a pure KCl tablet at various delay times ranging from 1 µs to 300 µs are shown in Figure 7. All K atomic emission features in both UV/Vis/NIR and LWIR ranges have lifetimes in the order of tens of micro-seconds. The LWIR emission bands due to the inter-high-excited-states transition of neutral potassium atoms decayed slightly faster than their UV/Vis/NIR counterparts. Under current experimental conditions, the first-excited-state-to-ground-state K atomic bands at 766 nm and 770 nm were still distinguishable in the UV/Vis/NIR LIBS spectrum even at 300 µs.

### 3.2. Evaluation of LWIR LIBS as an In Situ Chemical Sensor in Field Applications

For elements that are of most interest in field applications besides alkali metals, the UV/Visible/NIR LIBS-based probes generally have limits of detection (LOD) close to 1000 ppm or 0.1 weight percentage (% weight) [5]. To assess the molecular detection performance of LWIR LIBS in standoff detection at such target concentrations, five mixture samples of different acetaminophen concentration in a mineral (Al_2_O_3_) substrate were prepared for this study. Al_2_O_3_ is a main ingredient of many terrestrial soils and regoliths. The first sample is 25% weight acetaminophen +75% weight Al_2_O_3_, the second is 10% weight acetaminophen +90% weight Al_2_O_3_, the third is 1% weight acetaminophen +99% weight Al_2_O_3_, the fourth is 0.1% weight acetaminophen +99.9% weight Al_2_O_3_, and the fifth is a 100% Al_2_O_3_ sample tablet. The organic and inorganic materials in this study were ground carefully into fine powders and pressed into sample disks of 1 cm in diameter and 5 mm in thickness using a high-pressure hydraulic press. Each mixture sample studied was made into the same tablet form for measurements.

**Figure 8:**
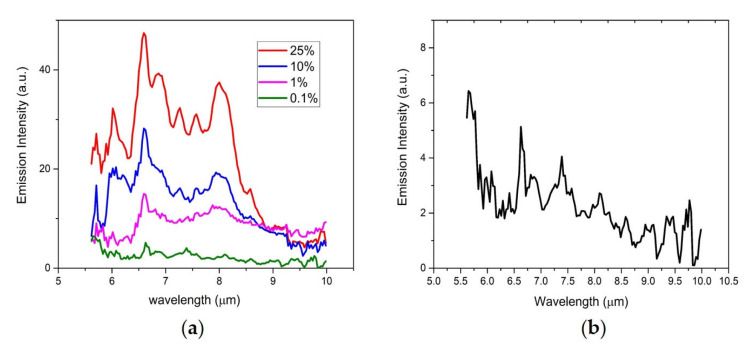
(**a**) LWIR spectra of four acetaminophen/Al_2_O_3_ mixture samples of different acetaminophen concentrations after removing the background substrate emissions. (**b**) LWIR LIBS spectra of the 0.1% weight acetaminophen/Al_2_O_3_ mixture sample after removing the background substrate emissions.

Figure 8a shows the integrated LWIR LIBS spectra of these mixture samples after removing the background and Al_2_O_3_ substrate emissions. Figure 8b shows the lowest one at 0.1% weight concentration (1000 ppm). The acetaminophen emission signatures listed in Table 1 could be consistently recognized in LWIR LIBS spectra of all four concentrations without the need of a more sophisticated analysis algorithm. It is reasonable to conclude that in this preliminary evaluation, the detection limit of this LWIR LIBS spectrometer with a linear MCT array detector should likely be ≤1000 ppm, which is similar to those LODs of UV/Vis/NIR LIBS-based probes NASA employed in both ChemCam and SuperCam [5,26,27].

**Figure 9:**
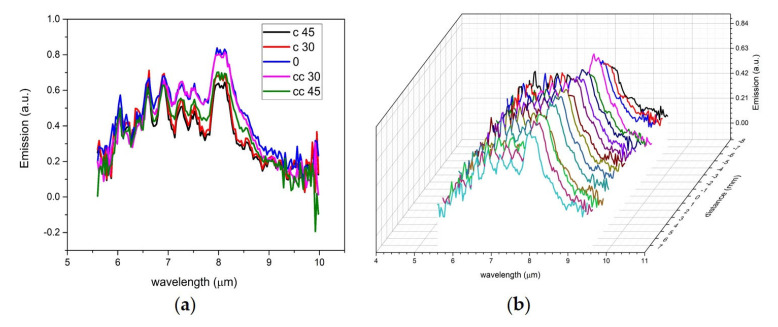
(**a**) LWIR LIBS spectra of acetaminophen at different sampling angles: 0 (parallel to the focal plane of the LWIR collection optics), c 30 (rotated 30° clockwise), c 45 (rotated 45° clockwise), cc 30 (rotated 30° counterclockwise), cc 45 (rotated 45° counterclockwise). (**b**) LWIR LIBS spectra of acetaminophen at different sampling distances. Distance 0 was set at one meter from the front end of the collection optics mirror set.

In order to evaluate the surface shape and roughness tolerance of a portable or mounted LWIR LIBS-based sensing instrument, we measured the LWIR LIBS spectra of acetaminophen tablets at different distances and angles. The acetaminophen sample tablets were measured starting from one meter away with an alignment parallel to the focal plane of the collection optics and the sample tablets were then gradually translated and rotated. The LWIR LIBS spectra of acetaminophen at different distances (Figure 9a) and angles (Figure 9b) showed a high degree of tolerance of sampling distance and angle variation. The LWIR LIBS spectra from 0.992 to 1.008 m and from −45 (rotating counterclockwise) to 45 degrees (rotating clockwise) are quite similar to each other, both qualitatively and quantitatively. All the major vibration emission signatures of acetaminophen (listed in Table 1) can be readily identified in all LWIR LIBS spectra with these distance and angle ranges.

### 3.3. LWIR LIBS Measurements Using a 2-D MCT FPA Detector

There are several ways to advance the LWIR LIBS detection scheme beyond the current 2 to 10 μm linear array system and further improve the understanding of hot neutral complex molecular gas in the vicinity of the laser-induced plasma. A high-performance 2-D FPA LWIR detector that can monitor both the spatial and temporal distribution of atomic, diatomic, and complex molecular LWIR-radiating plasma species will enable unprecedented experiments on fundamental mechanisms of laser-induced plasmas and shed light on the possible origins of those intact complex molecules in the vicinity of the laser-induced plasma plume. Because the emission lifetimes of molecular vibrational features in LWIR LIBS spectra generally last a few hundred micro-seconds, a 2-D FPA LWIR detector with the capability of collecting all the IR photons emitted by the plasma for more than 200 µs would result in a much-improved SNR of the captured spectrum. Extending the spectral range of the spectrometer to 12 μm will enable the detection of more chemical compounds such as many important organic and inorganic oxides. In the present work, LWIR LIBS spectra from solid samples were captured by a 2-D FPA LWIR detector, a low-dark-count broadband (2 to 12 μm) MCT FPA detector (PhaseTech, Madison, GA, USA), for the first time.

**Figure 10:**
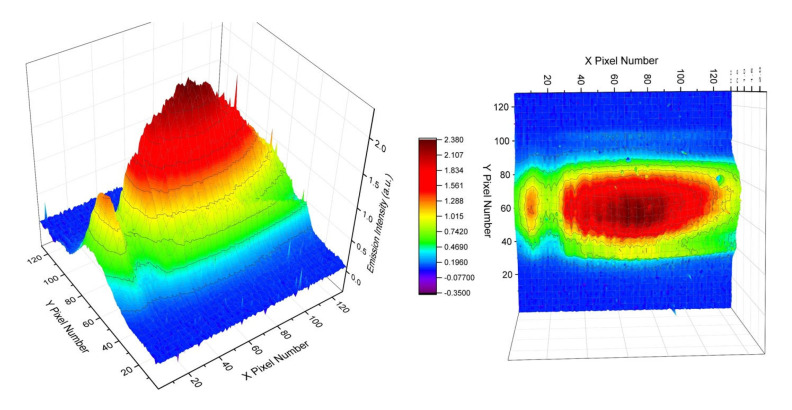
Infrared emission image from a 700 °C thermal radiation source on a PhaseTech 2−D detector array.

For spectral nonuniformity correction purposes [2,3], we first measured the infrared emissions from a 700 °C thermal radiation source (Boston Electronics (HelioWorks), Brookline, MA, USA) using the current collection optics setup couple with a PhaseTech 2-D MCT array detector. The monochromator is tuned to be centered at 7.5 um wavelength. The monochromator slit size is 0.5 mm and the integration time is 200 µs. The signal image is shown in Figure 10. The PhaseTech MCT 2-D array detector used in this study has a pixel format of 128 × 128. We labeled the direction parallel to the grating dispersion on the 2-D array with X pixel numbers (X coordinates) and the direction perpendicular to the grating dispersion with Y pixel numbers (Y coordinates). Each pixel is characterized by its X and Y coordinates.

The IR emission signals from the blackbody source centered on roughly row Y58 on the 2-D array detector with a full width at half maximum (FWHM) spanning about 34 pixels (sampling at row X70) in the direction perpendicular to the grating dispersion (Y-coordinate). The distance to the samples from the instrument, the front end of the collection optics, is around 1.03 m. A Cassegrain reflector-type telescope with a 6-inch primary mirror is used to collect emitted LWIR emission onto the input slit of the grating-based spectrometer. The magnification of the collection optics is ~0.67. The spectrometer uses a spherical concave mirror of 150 mm with a 50 mm focal length aspherical lens to focus the diffracted first order onto the FPA detector. The total magnification of the optical set is around 0.2217 or 1/4.5. The dimensions of our thermal radiation source are around 6.2 × 6.2 mm. Therefore, the image of the thermal radiation source on the detector array is about 1.377 mm in the direction that is perpendicular to the grating dispersion (Y coordinate). The PhaseTech MCT 2-D array detector has a 40 × 40 µm pitch size for each pixel and a 1.377 mm image on the detector corresponds to 34 pixels. Interestingly, the FWHM width of the LWIR signal along the Y coordinate roughly matched the image dimension of the entire thermal radiation source on the 2-D detector.

**Figure 11:**
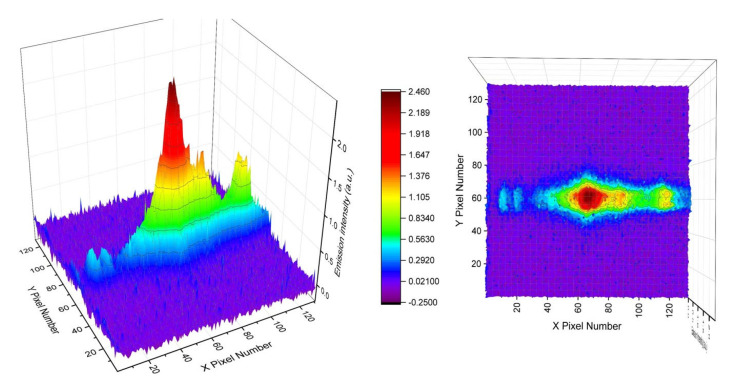
Single-shot LWIR LIBS emissions from KCl samples using the current collection optics setup and the PhaseTech 2−D array detector.

Similarly, we measured the LWIR LIBS emissions (Figure 11) from KCl samples using the current collection optics setup and PhaseTech 2-D MCT array detector. The excitation and collection operation setups were the same as current and previous studies using the linear MCT array detector. From earlier studies [3] and the current work described in Section 3.1, we learned that the LWIR LIBS emissions from a solid KCl sample tablet in ambient air atmosphere consist mainly of intense atomic emission features of K atoms. Additionally, the decay times of atomic emissions in the infrared region, similar to those in the UV–Visible region, are generally in the order of tens of micro-seconds. However, for an easier comparison with the molecular emissions, we set the integration time of the 2-D LWIR LIBS measurement of KCl as 200 µs and the delay time as 20 µs. The LWIR LIBS emissions of KCl, similar to those of acetaminophen, were centered near row Y59 on the 2-D array detector and with the FWHM spanning about 12 pixels (sampling at row X70) in the direction perpendicular to the grating dispersion (Y-coordinate). Using the pixel pitch size and the magnification factors of the current setup, we estimated the dimension of the atomic LWIR LIBS emissions from KCl to be around 2.1 mm along the axial direction perpendicular to the grating dispersion on the sample surface (i.e., perpendicular to the plane of incidence in the setup of this work, as shown in Figure 1). This size is sensible considering the typical dimension of the vapor–plasma plume of laser-induced plasma is in the order of millimeters [28].

**Figure 12:**
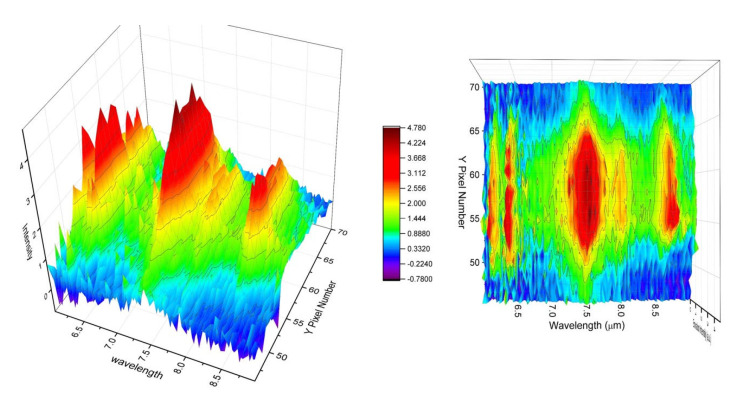
Calibration-corrected single-shot 2−D LWIR LIBS spectrum of KCl.

Similar to the calibration processes used on the linear array detector in current and previous studies [1,2,3,4], we performed pixel-by-pixel nonuniformity correction using the single-shot LWIR LIBS signal of a pixel and the corresponding blackbody signal of the corresponding pixel. All five distinctive atomic signature bands, 6P_1/2_ → 4D_3/2_, 6P_1/2_ → 6S_1/2_, 6H → 5G, 7S_1/2_ → 6P_3/2_, and 5D_5/2_ → 6P_3/2_, of the K atom could be identified in the spectrum. For wavelength calibration, the dispersion function of this 2-D detector setup was formulated by using the center wavelengths of these five bands as wavelength markers. The nonuniformity- and wavelength-corrected 2-D LWIR LIBS spectrum of KCl is shown in Figure 12.

**Figure 13:**
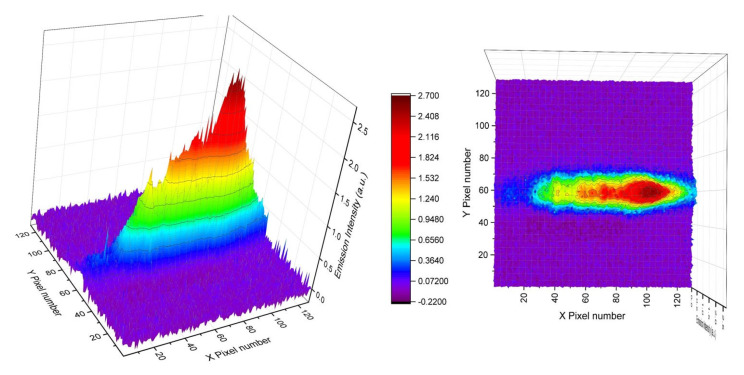
Single-shot LWIR LIBS emissions from acetaminophen tablets using the current collection optics setup and the PhaseTech 2-D array detector.

Next, we measured the LWIR LIBS emissions from acetaminophen sample tablets using the current collection optics setup and the PhaseTech 2-D array detector. The operation parameters and setups were mostly the same as previous studies using the linear MCT array detector. The only difference is that the linear array detector is replaced with the PhaseTech 2-D MCT array detector. This 2-D IR array detector has lower dark counts than the linear MCT array we currently employed. Therefore, the integration time of the 2-D LWIR LIBS measurement was set to be 200 µs to capture long lifetime LWIR LIBS molecular emissions. The whole 2-D image of the IR emissions from single-shot laser-induced plasma generated on the surfaces of the acetaminophen samples with a 20 µs delay and 200 µs integration window is plotted in Figure 13.

**Figure 14:**
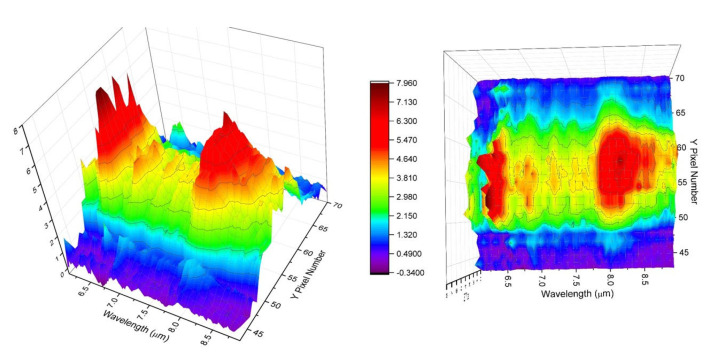
Calibration-corrected single-shot 2−D LWIR LIBS spectrum of acetaminophen.

The LWIR LIBS emissions of acetaminophen were centered near row Y58 on the 2-D array detector with the FWHM spanning about 14 pixels (sampling at row X70) in the direction perpendicular to the grating dispersion (Y-coordinate). Again, using the pixel pitch size and the magnification factors of the current setup, we estimated the dimension of the molecular LWIR LIBS emissions from acetaminophen to be around 2.5 mm on the sample surface along the axial direction perpendicular to the grating dispersion (i.e., perpendicular to the plane of incidence). The nonuniformity- and wavelength-corrected 2-D spectrum of acetaminophen LWIR LIBS emissions is shown in Figure 14. All major vibrational signature emissions of acetaminophen in the 6–9 µm spectral range (Table 1) were clearly observed.

**Figure 15:**
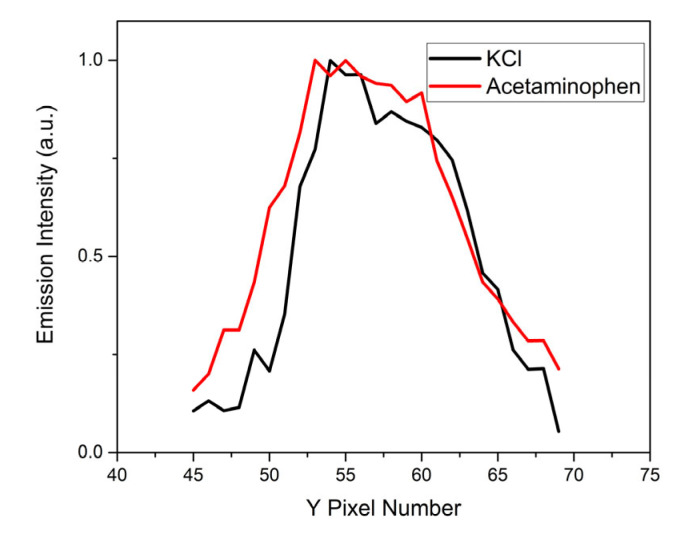
Calibration-corrected LWIR LIBS emission profiles of KCl (black) and acetaminophen (red) in the direction perpendicular to the grating dispersion.

This 2-D detection setup offers the detection system the capability of resolving the spatial distribution of the LWIR emitters in and near the vapor–plasma plume. From Figure 12 and Figure 14, we can see the spatial distribution of the LWIR LIBS spectrum along the direction perpendicular to the dispersion (Y coordinate). The LWIR LIBS spectrum of each row comes from emitters located at different axial positions along the direction perpendicular to the plane of incidence on the sample surface. We can readily see the variations of the spectral patterns as a function of the positions in and near the plasma plume. Calibration-corrected LWIR LIBS emission profiles of the KCl 5D_5/2_ → 6P_3/2_ band at 8.53 µm and the acetaminophen CO stretching + Aromatic CC stretching band at 8 µm along the direction perpendicular to the grating dispersion (Y coordinate) are shown in Figure 15. Comparing the spatial profiles of the calibration-corrected LWIR LIBS emissions between KCl and acetaminophen, the spatial distribution of atomic emitters (K atoms) and molecular emitters (intact acetaminophen molecules) are comparable, in general, and exhibit small differences. The LWIR signature emissions from both atomic emitters and molecular emitters were imaged on the FPA detector mainly between row Y45 and row Y70. The spatial distribution of intact molecular emitters is slightly broader than their atomic counterparts. This preliminary result indicated that the observed LWIR intact-target-molecular emitters is likely located in the vicinity of the highly excited LWIR atomic emitters in the laser-induced vapor–plasma plume.

**Figure 16:**
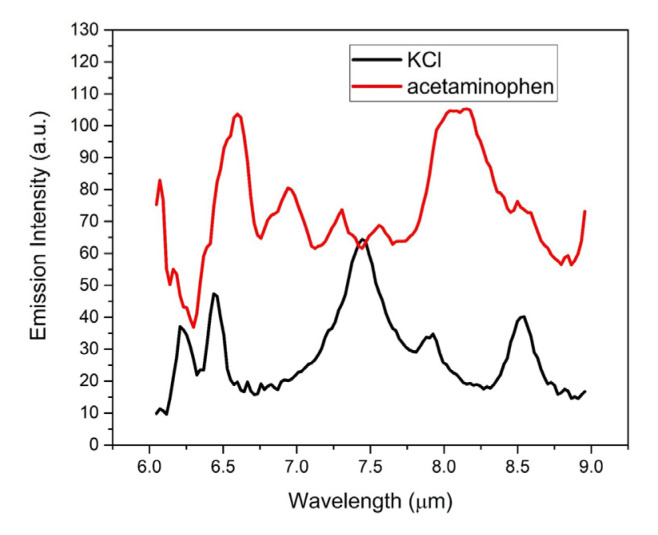
Single-shot LWIR LIBS spectra of acetaminophen (red) and KCl (black) after integrating all 26 individual spectra from row Y45 to row Y70.

Finally, Figure 16 shows the LWIR LIBS spectra of acetaminophen and KCl after integrating all 26 individual spectra of each row from row Y45 to row Y70. When integrated both temporally (≥200 µs) and spatially, the LWIR LIBS emissions from intact molecular species apparently have similar signature emission intensities and SNRs as those from the atomic emitters. The intensity of the acetaminophen CC stretching band at 6.62 µm is roughly the same as the 6H → 5G band intensity of K atoms at 7.43 µm. In comparison, the 6.62 µm band intensity of acetaminophen was only about 6% of the 7.43 µm band intensity of K atoms in single-shot spectra of the current 1-D detection setup. That relative intensity throughput (ratio of the 6.62 µm acetaminophen band intensity to the 7.43 µm K atomic band intensity) of 2-D measurements was, thus, improved by almost 16 times compared to those measured by a 1-D detector. The SNRs of the 6.62 µm band of the acetaminophen molecule measured in 1-D and 2-D detection setups were 6.1 and 22.8, respectively. The SNRs of the 7.43 µm band of K atoms measured in 1-D and 2-D detection setups were 15.9 and 18.1, respectively. While the 2-D setup improved the SNR of LWIR atomic measurements marginally, it enhanced the molecular emission measurements by a factor of 3.73. A larger signal-to-noise ratio will result in lower detection limits [29]. Therefore, with significant enhancements of both signal intensities and SNRs by employing a 2-D MCT array detector in the current experimental setup, the LWIR LIBS detection system significantly improved its capability of detecting a trace amount of target materials and, thus, showed great promise to complement UV/Vis/NIR LIBS and perform real-time chemical–biological detection. Future systematic optimizations on the collection optics and experimental parameters for the 2-D FPA detector will further enhance the SNR and, thus, lower the detection limit of LWIR LIBS estimated using the linear array detector. More rigorous detection limit evaluations will be attempted during the system optimizations in the near future.

## 4. Conclusions

Studies of LWIR emissions from laser-induced plasma have established the presence of a related phenomenon, LWIR LIBS. In LWIR LIBS, the molecular structure of constituents is revealed by spectrally analyzing the emission profile at a delayed time gate from the continuum emission of the LIBS plasma using long-wave infrared spectrometry. This pioneering research has effectively introduced a new field of analytical science to the LIBS community, affording unique molecular analysis capabilities that would be intractable using standard infrared spectroscopy.

As demonstrated in this work, LWIR LIBS can acquire distinct molecular vibrational signatures in a broadband infrared spectrum within micro-seconds after the plasma initiation. The detection limit of the LWIR LIBS spectrometer with a linear MCT array detector employed in this work was estimated to be ≤1000 ppm, which is similar to those of the UV/Vis/NIR LIBS probes currently employed on NASA’s ChemCam and SuperCam. Due to the spatial distribution (in the order of millimeters) and long lifespan (in the order of hundreds of microseconds) of the LWIR emitters in the vicinity of the laser-induced plasma, the signal intensities and SNRs of the LWIR LIBS-based detection system were considerably improved by employing a low dark-count 2-D MCT FPA detector. The use of the 2-D MCT FPA detector in the LWIR LIBS spectrometer not only improves the detection sensitivities, but also adds the capability of revealing spatial distributions of the LWIR emissions from laser-induced plasma. Further optimization of the measurements of laser-induced plasma emissions with a 2-D MCT FPA detector to improve the signal throughput, detection limits, and spectral ranges will open up new possibilities for LWIR LIBS development.

By measuring the LWIR LIBS spectra of acetaminophen at various sample distances and tilting angles, we estimate that the quality of the spectra remains relatively unchanged within the range of a 1 m ± 0.8 cm sampling distance and a ± 45 degree perpendicular tilting axis. This high degree of sample alignment tolerance makes standoff sensor based on LWIR LIBS spectroscopy attractive in field applications that prefer a technique with intense signal responses and that is affected very little by the surface shape, roughness, and composition irregularity. By pairing with conventional ultraviolet–visible–near infrared (UV/Vis/NIR LIBS) LIBS, simultaneous UV/Vis/NIR LIBS + LWIR LIBS promises unprecedented capability of in situ, real-time, and stand-off investigations of both atomic and molecular target compositions to detect and characterize a range of chemistries. With its capabilities of rapidly and precisely identifying the contaminants in a warzone or hazardous civilian area, this UV/Vis/NIR LIBS + LWIR LIBS analytical approach has the potential to solve numerous problems such as the rapid detection of trace explosives and hazardous compounds, including chemical warfare agents, toxic industrial materials, and pharmaceutical compounds, on operational surfaces.

## Figures and Tables

**Table 1:** Vibrational mode assignments of the acetaminophen LWIR LIBS bands.

DFT Calculation (µm)	Assignment	LWIR LIBS (µm)
5.92	C = O stretching	6
6.2	Aromatic CC stretching	6.17
6.31	Aromatic CC stretching + CNH bending	6.44
6.65	Aromatic CC stretching	6.62
6.89	CH_3_ bending + Aromatic CC stretching + CNH bending + COH bending	6.83
6.96	CH_3_ bending + Aromatic CC stretching + CNH bending + COH bending	6.95
7.2	NH bending + Aromatic CC stretching	7.25
7.44	CH_3_ bending + CCN stretching	7.52
7.97	CO stretching + Aromatic CC stretching	7.95
8.22	CN stretching + Aromatic CH bending	8.1
8.62	OH bending + Aromatic CH bending	8.57
9.14	OH bending + Aromatic CH bending	9.11

## Data Availability

Not applicable.

## References

[1] Bower D.M., Yang C.S.C., Hewagama T., Nixon C.A., Aslam S., Whelley P.L., Eigenbrode J.L., Jin F., Ruliffson J., Kolasinski J.R. (2021). Spectroscopic characterization of samples from different environments in a Volcano-Glacial region in Iceland: Implications for in situ planetary exploration. Spectrochim. Acta A Mol. Biomol. Spectrosc..

[2] Yang C.S., Bower D.M., Jin F., Hewagama T., Aslam S., Nixon C.A., Kolasinski J.R., Samuels A.C. (2022). Raman and UVN+LWIR LIBS detection system for in-situ surface chemical identification. MethodsX.

[3] Yang C.S.-C., Brown E., Kumi-Barimah E., Hommerich U., Jin F., Trivedi S.B., Samuels A.C. (2015). Rapid long-wave infrared laser-induced breakdown spectroscopy measurements using a mercury-cadmium-telluride linear array detection system. Appl. Opt..

[4] Yang C.S.-C., Jin F., Trivedi S.B., Brown E., Hommerich U., Nemes L., Samuels A.C. (2019). In situ chemical analysis of geology samples by a rapid simultaneous ultraviolet/visible/near-infrared (UVN) + longwave-infrared laser induced breakdown spectroscopy detection system at standoff distance. Opt. Express.

[5] Clegg S., Anderson R., Melikechi N., Bishop J., Bell J., Moersch J. (2019). Laser-induced breakdown spectroscopy: Theory and laboratory spectra of geologic materials. Remote Compositional Analysis: Techniques for Understanding Spectroscopy, Mineralogy, and Geochemistry of Planetary Surfaces.

[6] Hahn D.W., Omenetto N. (2010). Laser-induced breakdown spectroscopy (LIBS), part I: Review of basic diagnostics and plasma-particle interactions: Still-challenging issues within the analytical plasma community. Appl. Spectrosc..

[7] Yu X., Li Y., Gu X., Bao J., Yang H., Sun L. (2014). Laser-induced breakdown spectroscopy application in environmental monitoring of water quality: A review. Environ. Monit. Assess..

[8] Gonçalves D.A., Senesi G.S., Nicolodelli G. (2021). Laser-induced breakdown spectroscopy applied to environmental systems and their potential contaminants. An overview of advances achieved in the last few years. Trends Environ. Anal. Chem..

[9] Yang C.S.-C., Jin F., Trivedi S.B., Brown E., Hommerich U., Tripathi A., Samuels A.C. (2017). Long-wave infrared (LWIR) molecular laser-induced breakdown spectroscopy (LIBS) emissions of thin solid explosive powder films deposited on aluminum substrates. Appl. Spectrosc..

[10] Yang C.S.-C., Jin F., Trivedi S.B., Swaminathan S.R., Patel S., Ramer E.D., Brown E., Hommerich U., Samuels A.C. (2017). Comprehensive study of solid pharmaceutical tablets in visible, near infrared (NIR), and longwave infrared (LWIR) spectral regions using a rapid simultaneous ultraviolet/visible/NIR (UVN) + LWIR laser-induced breakdown spectroscopy linear arrays detection system and a fast acousto-optic tunable filter NIR spectrometer. Opt. Express.

[11] Haddad J.E., Canioni L., Bousquet B. (2014). Good practices in LIBS analysis: Review and advices. Spectrochim. Acta Part B: Atomic Spectrosc..

[12] Bilge G., Eseller K.E., Berberoglu H., Sezer B., Tamer U., Boyaci I.H. (2021). Comparison of different calibration techniques of laser induced breakdown spectroscopy in bakery products: On NaCl measurement. J. Eur. Opt. Soc.-Rapid Publ..

[13] Han W., Wang Y., Yin Y., Li X., Sun D., Su M. (2022). Analysis of metal elements contained in graphite target coated with Chinese medicinal material nanoparticles using LIBS. Front. Phys..

[14] Moros J., Laserna J. (2019). Laser-induced breakdown spectroscopy (LIBS) of organic compounds: A review. Appl. Spectrosc..

[15] Xu F., Ma S., Zhao C., Dong D. (2022). Application of molecular emissions in laser-induced breakdown spectroscopy: A review. Front. Phys..

[16] Amado A.M., Azevedo C., Ribeiro-Claro P.J.A. (2017). Conformational and vibrational reassessment of solid paracetamol. Spectrochim. Acta A Mol. Biomol. Spectrosc..

[17] Friesner R.A. (2005). Ab initio quantum chemistry: Methodology and applications. Proc. Natl. Acad. Sci. USA.

[18] Neese F., Wennmohs F., Becker U., Riplinger C. (2020). The ORCA quantum chemistry program package. J. Chem. Phys..

[19] Zeinalipour-Yazdi C.D. (2022). A DFT study of the interaction of aspirin, paracetamol and caffeine with one water molecule. J. Mol. Model..

[20] Burgina E.B., Baltakhinov V.P., Boldyreva E.V., Shakhtschneider T.P. (2004). IR spectra of paracetamol and phenacetin. 1. Theoretical and experimental studies. J. Struct. Chem..

[21] Colthup N.B. (1980). Molecular orbitals and CH_3_, CH_2_, and CH deformation group frequencies. Appl. Spectrosc..

[22] Asamowa E., Hongbing Y. (2017). Influence of laser energy on the electron temperature of a laser-induced Mg plasma. Appl. Phys. B.

[23] Ralchenko Y., Kramida A., Reader J. NIST ASD Team 2011, NIST Atomic Spectra Database (version 4.1.0). https://www.nist.gov/pml/atomic-spectra-database.

[24] Rice S.A., Klemperer W. (1957). Spectra of the alkali halides. II. The infrared spectra of the sodium and potassium halides, RbCl, and CsCl. J. Chem. Phys..

[25] Civis S., Ferus M., Kubelik P., Jelinek P., Chernov V.E. (2012). Potassium spectra in the 700–7000 cm^−1^ domain: Transitions involving f-, g-, and h-states. Astron. Astrophys..

[26] Wiens R.C., Maurice S., Robinson S.H., Nelson A.E., Cais P., Bernardi P., Newell R.T., Clegg S., Sharma S.K., Storms S. (2021). The SuperCam instrument suite on the NASA Mars 2020 rover: Body unit and combined system tests. Space Sci. Rev..

[27] Wiens R.C., Maurice S., Lasue J., Forni O., Anderson R.B., Clegg S., Benderd S., Blaneye D., Barracloughd B.L., Cousinab A. (2013). Pre-flight calibration and initial data processing for the ChemCam laser-induced breakdown spectroscopy instrument on the Mars Science Laboratory rover. Spectrochim. Acta Part B: Atomic Spectrosc..

[28] Shi Z., Hardalupas Y., Taylor A.M.K.P. (2019). Laser-induced plasma image velocimetry. Exp. Fluids.

[29] Hahn D.W., Omenetto N. (2012). Laser-induced breakdown spectroscopy (LIBS), part II: Review of instrumental and methodological approaches to material analysis and applications to different fields. Appl. Spectrosc..

